# Supplementary material for: Modeling transcriptional regulation of the cell cycle using a novel cybernetic-inspired approach
Source: Biophys J. 2023 Dec 15;123(2):221–34. doi: 10.1016/j.bpj.2023.12.010 (PMC10808046; doi:10.1016/j.bpj.2023.12.010)
Supplement: Document S1. Figures S1–S7 and Tables S1 and S2 [file mmc1.pdf]

Biophysical Journal, Volume *123*

Supplemental information

Modeling transcriptional regulation of the cell cycle using a novel cybernetic-inspired approach

Rubesh Raja, Sana Khanum, Lina Aboulmouna, Mano R. Maurya, Shakti Gupta, Shankar Subramaniam, and Doraiswami Ramkrishna

SUPPORTING INFORMATION:

Modeling transcriptional regulation of the cell cycle using a novel cybernetic-inspired approach

Rubesh Raja[1], Sana Khanum[1], Lina Aboulmouna[2], Mano R. Maurya[2], Shakti Gupta[2], Shankar Subramaniam[2,3,*] and Doraiswami Ramkrishna[1,*]

[1]The Davidson School of Chemical Engineering, Purdue University, West Lafayette, IN 47907, USA; raja11@purdue.edu (R.R.); khanum@purdue.edu (S. K.)

[2]Department of Bioengineering, University of California San Diego, La Jolla, CA 92093, USA; laboulmouna@ucsd.edu (L.A.); mano@sdsc.edu (M.R.M.); shgupta@ucsd.edu (S.G.)

[3]Departments of Computer Science and Engineering, Cellular and Molecular Medicine, San Diego Supercomputer Center, and the Graduate Program in Bioinformatics and Systems Biology, University of California San Diego, La Jolla, CA 92093, USA

Table S1: Cybernetic model RNA species-agnostic parameters

Parameters	Value	Unit
α	0.7705	g-unit/h
β	0.6349	h^{-1}
f_{G0}	0.3091	-
t_1	9.2511	h
t_2	14.4324	h
t_3	17.5751	h
t_4	23.7185	h
t_5	32.4408	h
t_6	40.2104	h
t_7	49.8328	h
t_8	51.6155	h

Here, ‘g-unit’ is the basic unit of the regulator of gene expression (*g*).

Table S2: Cybernetic model RNA species-specific parameters

Parameter<br>Unit	k_j^r<br>h^{-1} g-unit^{-1}	$g_j(0)$<br>g-unit	γ_j<br>h^{-1}	$w_{G1,j}$<br>-	$w_{S1,j}$<br>-	$w_{S2,j}$<br>-	$w_{S3,j}$<br>-	$w_{G2,j}$<br>-	$w_{M,j}$<br>-
Abl1	2.070	0.920	1.036	0.016	0.014	0.014	0.012	0.012	0.010
Anapc1	1.701	1.070	0.654	0.013	0.009	0.011	0.012	0.012	0.016
Atm	1.895	0.378	0.699	0.011	0.013	0.014	0.010	0.011	0.017
Bub1	1.914	0.316	0.337	0.004	0.009	0.021	0.020	0.017	0.024
Bub1b	2.163	0.316	0.424	0.005	0.005	0.014	0.017	0.013	0.020
Bub3	2.581	1.283	0.675	0.014	0.012	0.012	0.014	0.012	0.010
Ccnb2	2.009	0.447	0.318	0.004	0.002	0.011	0.017	0.014	0.016
Ccnd1	1.869	1.781	0.489	0.015	0.014	0.013	0.020	0.023	0.013
Ccne1	2.689	0.316	0.316	0.006	0.026	0.014	0.010	0.012	0.005
Ccnh	1.487	1.251	0.343	0.015	0.011	0.009	0.009	0.011	0.010
Cdc14b	2.140	1.525	0.888	0.010	0.008	0.008	0.009	0.009	0.012
Cdc20	1.537	0.317	0.316	0.015	0.012	0.036	0.038	0.041	0.026
Cdc25a	1.664	2.039	0.331	0.015	0.016	0.011	0.011	0.011	0.005
Cdc25b	1.658	0.317	0.412	0.009	0.008	0.014	0.021	0.013	0.021
Cdc45	1.538	0.329	0.316	0.013	0.024	0.016	0.014	0.017	0.011
Cdc6	4.973	0.316	0.547	0.004	0.015	0.008	0.005	0.009	0.004
Cdc7	2.267	0.317	1.111	0.011	0.017	0.020	0.017	0.016	0.021
Cdh1	0.768	1.842	0.464	0.017	0.015	0.005	0.007	0.007	0.011
Cdk1	1.740	0.316	0.316	0.012	0.015	0.014	0.014	0.015	0.015
Cdk2	1.819	1.789	0.316	0.010	0.017	0.011	0.009	0.010	0.009
Cdk4	1.560	1.697	0.316	0.015	0.017	0.012	0.013	0.013	0.010
Cdkn1a	3.623	2.574	1.647	0.015	0.008	0.006	0.007	0.007	0.005
Cdkn1b	1.451	0.316	0.554	0.011	0.006	0.011	0.009	0.009	0.018
Cdkn2a	1.312	1.014	0.316	0.011	0.009	0.011	0.012	0.013	0.010
Cdkn2b	1.440	4.966	0.403	0.008	0.008	0.008	0.011	0.012	0.018
Cdkn2c	1.511	0.319	0.363	0.006	0.011	0.017	0.015	0.011	0.020
Cdkn2d	2.403	0.317	0.615	0.006	0.007	0.020	0.022	0.014	0.025
Chek1	1.610	0.430	0.323	0.009	0.017	0.017	0.014	0.019	0.015
Crebbp	1.548	0.609	0.584	0.012	0.006	0.007	0.006	0.007	0.012
Dbf4	1.797	1.139	0.319	0.010	0.012	0.019	0.020	0.018	0.019
E2f1	1.800	0.316	0.417	0.009	0.021	0.013	0.011	0.014	0.009
E2f4	1.701	2.419	0.407	0.013	0.012	0.009	0.009	0.009	0.005
Espl1	2.573	0.317	0.650	0.006	0.010	0.021	0.024	0.019	0.023
Gadd45a	0.381	1.119	0.469	0.007	0.011	0.016	0.013	0.018	0.021
Gsk3b	1.174	0.495	0.320	0.010	0.007	0.008	0.007	0.009	0.014
Hdac2	1.694	1.443	0.460	0.012	0.010	0.010	0.009	0.011	0.009
Mad1l1	1.968	1.148	0.538	0.017	0.019	0.016	0.020	0.019	0.014
Mad2l1	2.065	0.318	0.316	0.007	0.016	0.024	0.022	0.021	0.019
Mcm3	2.790	0.317	0.529	0.011	0.021	0.014	0.012	0.016	0.009
Mdm2	5.074	2.653	4.521	0.019	0.012	0.013	0.013	0.014	0.012
Myc	10.011	3.515	4.803	0.009	0.006	0.003	0.004	0.004	0.001
Orc1	2.559	0.368	0.453	0.020	0.026	0.019	0.019	0.021	0.014

Pcna	1.886	1.928	0.325	0.013	0.023	0.013	0.009	0.013	0.010
Pkmyt1	1.582	0.316	0.317	0.009	0.020	0.015	0.015	0.015	0.013
Plk1	2.185	0.319	0.354	0.007	0.007	0.021	0.031	0.019	0.021
Prkdc	1.186	0.318	0.471	0.007	0.008	0.011	0.010	0.013	0.020
Pttg1	1.698	7.375	0.339	0.002	0.008	0.016	0.024	0.019	0.023
Rad21	1.350	0.621	0.316	0.013	0.009	0.016	0.014	0.013	0.019
Rb1	1.264	0.525	0.317	0.010	0.007	0.011	0.007	0.008	0.015
Rbl1	2.506	0.317	0.750	0.011	0.013	0.019	0.013	0.012	0.020
Sfn	0.644	6.667	0.321	0.024	0.031	0.008	0.011	0.007	0.000
Skp2	2.932	0.316	1.161	0.013	0.014	0.015	0.015	0.017	0.013
Smad2	1.596	1.336	0.366	0.011	0.010	0.008	0.007	0.008	0.008
Smad4	1.629	1.904	0.317	0.011	0.006	0.005	0.006	0.006	0.008
Smc1a	1.435	1.810	0.318	0.009	0.010	0.010	0.010	0.010	0.011
Smc3	2.335	1.235	0.911	0.013	0.010	0.010	0.009	0.011	0.013
Stag1	1.414	0.559	0.380	0.009	0.006	0.008	0.008	0.008	0.011
Tfdp1	3.729	1.025	1.674	0.017	0.015	0.015	0.014	0.015	0.013
Tgfb1	4.087	0.903	0.703	0.021	0.008	0.002	0.005	0.005	0.000
Trp53	1.989	1.762	0.326	0.010	0.011	0.007	0.008	0.009	0.005
Ttk	2.397	0.316	0.634	0.005	0.009	0.020	0.019	0.016	0.025
Wee1	2.458	0.771	0.594	0.009	0.012	0.013	0.014	0.011	0.012
Zbtb17	2.375	2.232	0.643	0.013	0.009	0.006	0.008	0.008	0.006
Arntl	3.121	1.192	0.691	0.021	0.010	0.004	0.004	0.006	0.003
Batf3	1.349	3.658	0.396	0.005	0.012	0.010	0.007	0.009	0.006
Brca1	2.260	0.317	0.511	0.006	0.016	0.018	0.012	0.014	0.022
Cebpb	6.628	2.775	2.950	0.011	0.004	0.002	0.003	0.004	0.003
Cux1	2.357	1.027	0.714	0.015	0.013	0.014	0.013	0.013	0.012
E2f7	2.143	0.316	0.361	0.011	0.025	0.021	0.017	0.020	0.014
Ets1	4.454	2.226	1.096	0.010	0.007	0.003	0.004	0.004	0.003
Foxm1	2.006	0.323	0.344	0.006	0.013	0.024	0.024	0.020	0.019
Foxo1	1.306	3.130	0.492	0.015	0.011	0.009	0.010	0.008	0.007
Gabpb1	3.197	3.034	1.140	0.015	0.009	0.006	0.006	0.006	0.006
Klf4	2.238	9.998	5.455	0.011	0.005	0.002	0.002	0.002	0.003
Lef1	1.960	2.579	0.598	0.014	0.015	0.008	0.009	0.008	0.005
Nfatc1	3.504	7.366	0.957	0.014	0.005	0.002	0.003	0.003	0.003
Nfkb1	2.596	2.767	0.819	0.012	0.006	0.003	0.004	0.004	0.003
Notch1	2.197	3.132	1.693	0.012	0.008	0.004	0.004	0.004	0.003
Prdm1	9.708	3.003	0.576	0.005	0.002	0.001	0.001	0.001	0.001
Rela	6.312	2.780	3.244	0.009	0.005	0.004	0.004	0.004	0.003
Relb	6.405	3.352	5.961	0.014	0.008	0.005	0.005	0.006	0.004
Rfx1	1.791	1.358	0.423	0.012	0.009	0.009	0.010	0.010	0.012
Rorb	1.536	0.575	0.377	0.039	0.020	0.009	0.007	0.008	0.004
Runx1	1.710	4.030	0.435	0.020	0.007	0.011	0.007	0.006	0.005
Sox2	4.732	0.576	3.680	0.017	0.009	0.008	0.010	0.010	0.007
Vdr	10.034	0.743	2.026	0.004	0.003	0.003	0.003	0.004	0.006

Here, ‘g-unit’ is the basic unit of the regulator of gene expression (g).

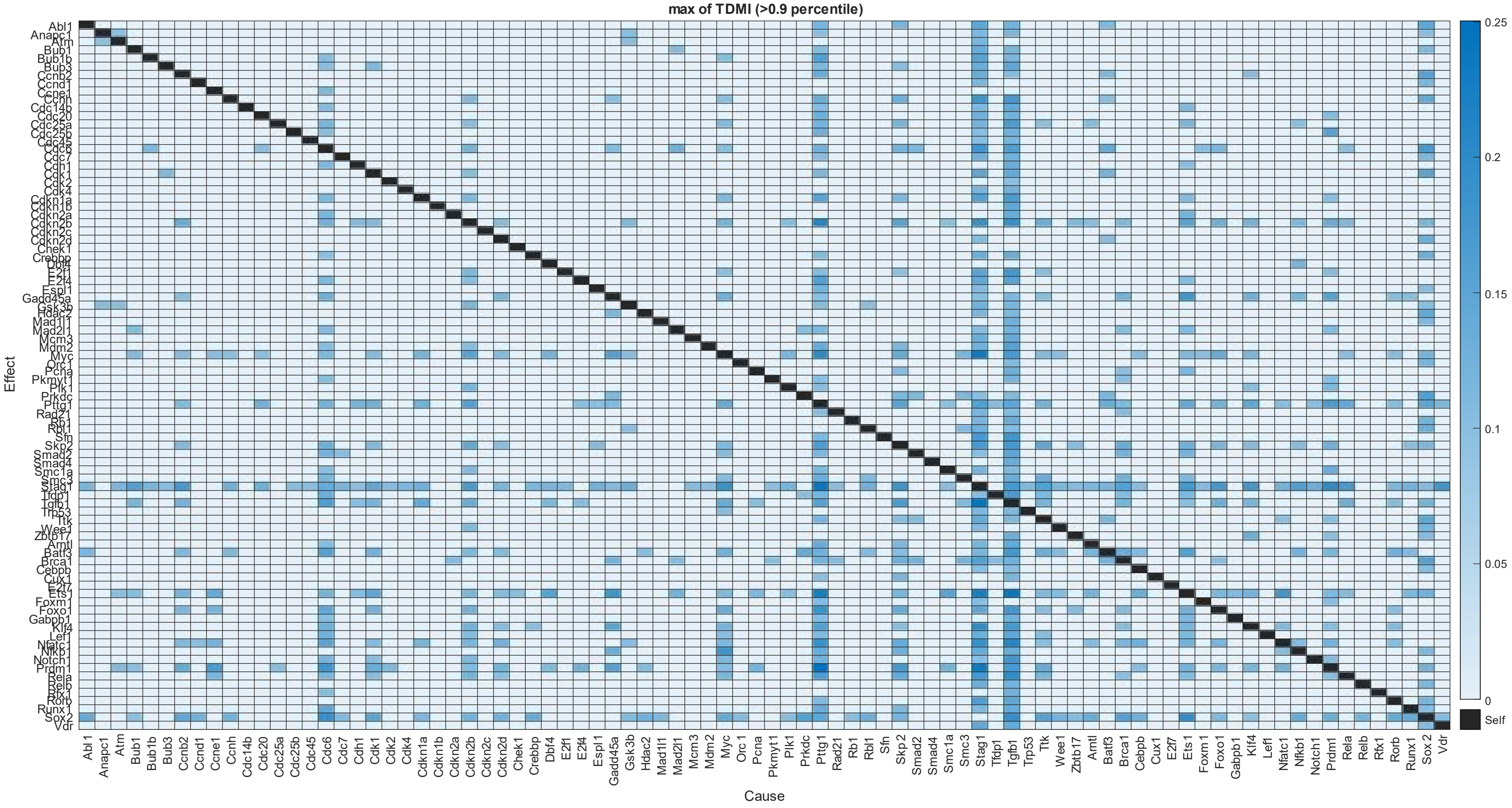

Figure S1: Development of interaction network using TDMI values. The heatmap shows the maximum TDMI value between two RNAs across different delays. Only interactions with TDMI of top 0.1 of the TDMI values are shown.

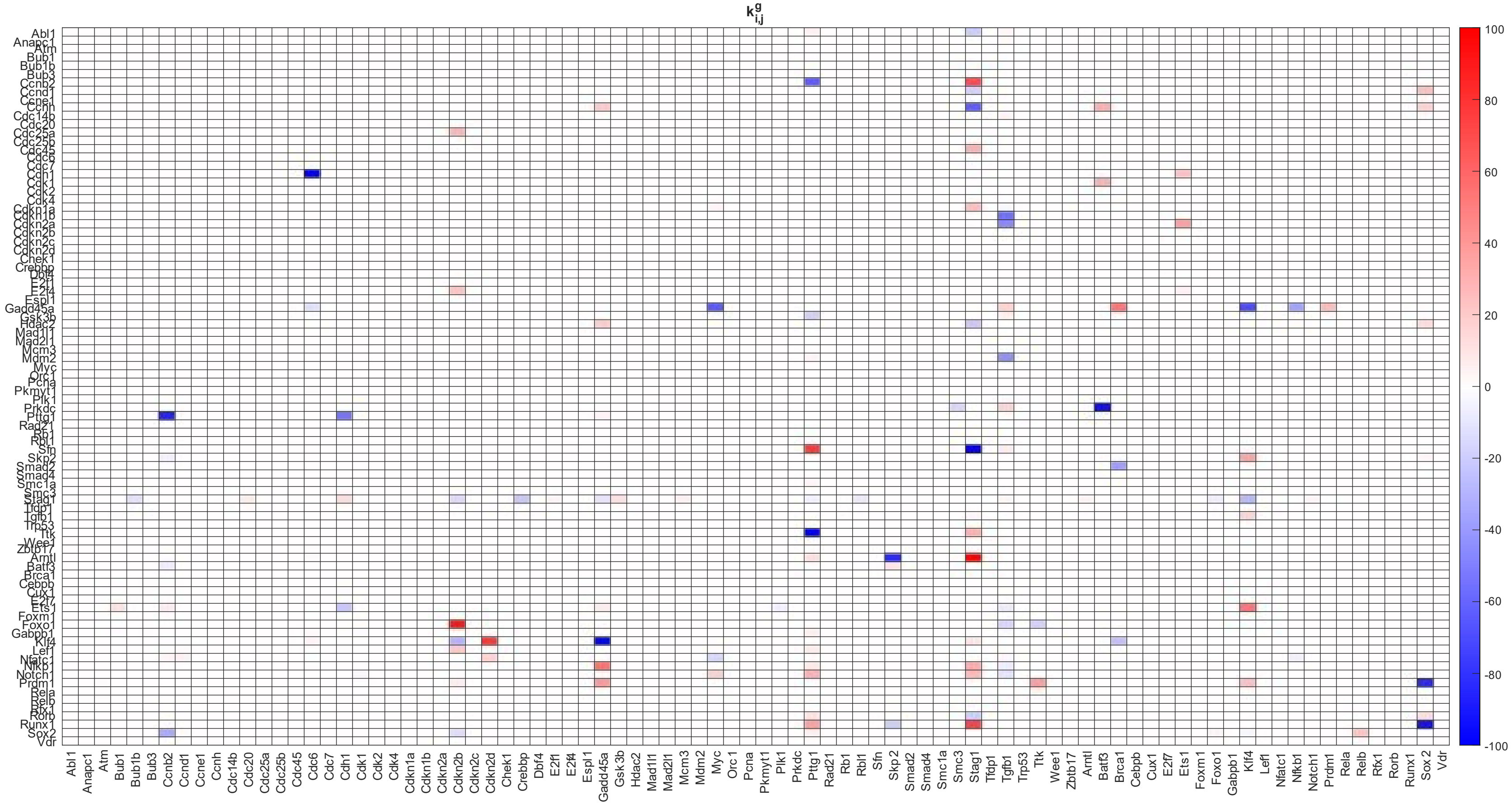

Figure S2: Heat map for the interaction parameter $k^g_{i,j}$. Red color represents activation interaction and blue color represents repressive interaction.

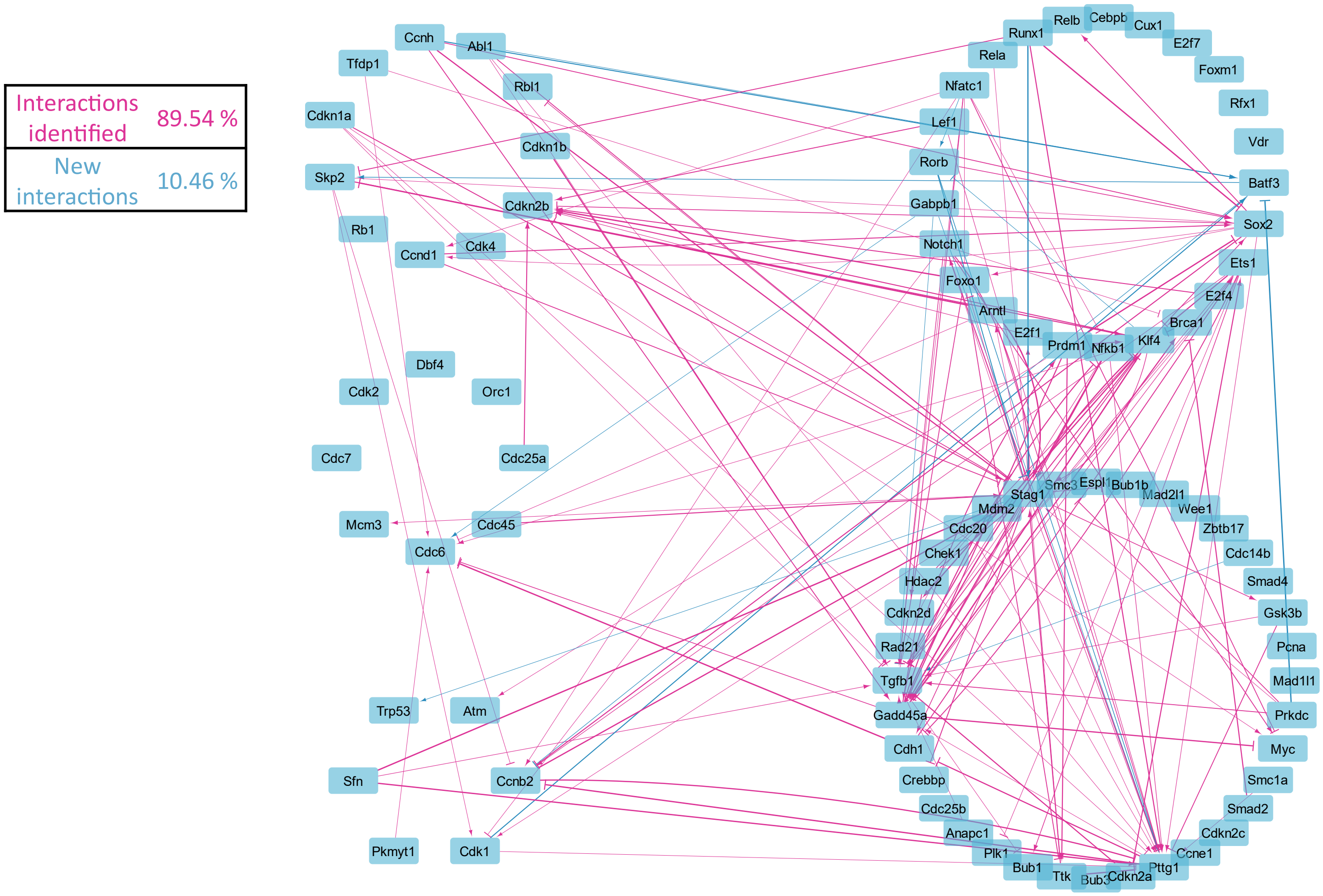

Figure S3: Comparing our network with String database. Comparing the network with a canonical protein-protein interaction network including second neighbor interactions shows 90% of these interactions are validated.

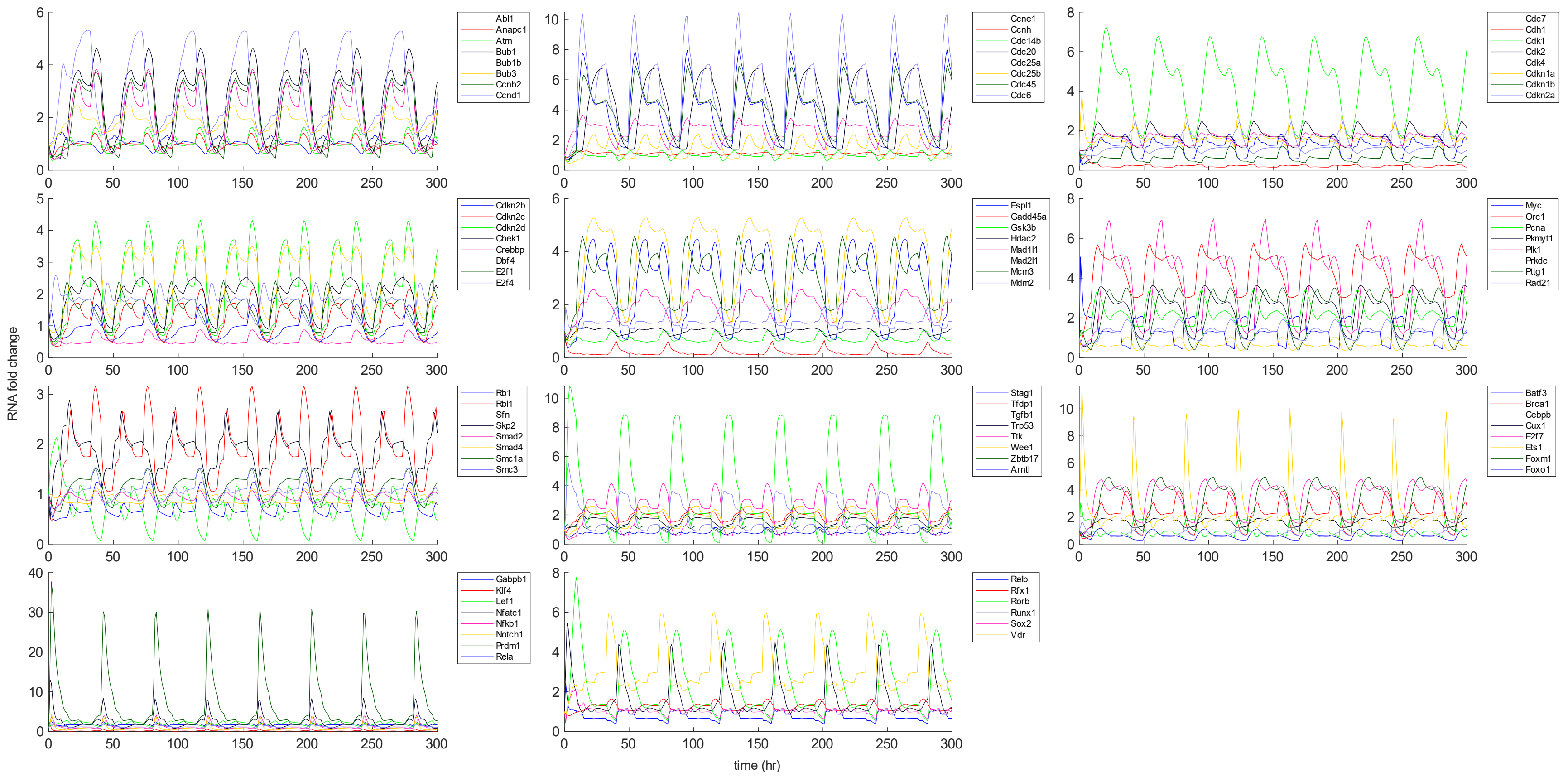

Figure S4: Cyclicity in our model. The figure shows that if we repeat the objectives for each phase in the same order, the model shows a cyclic behavior.

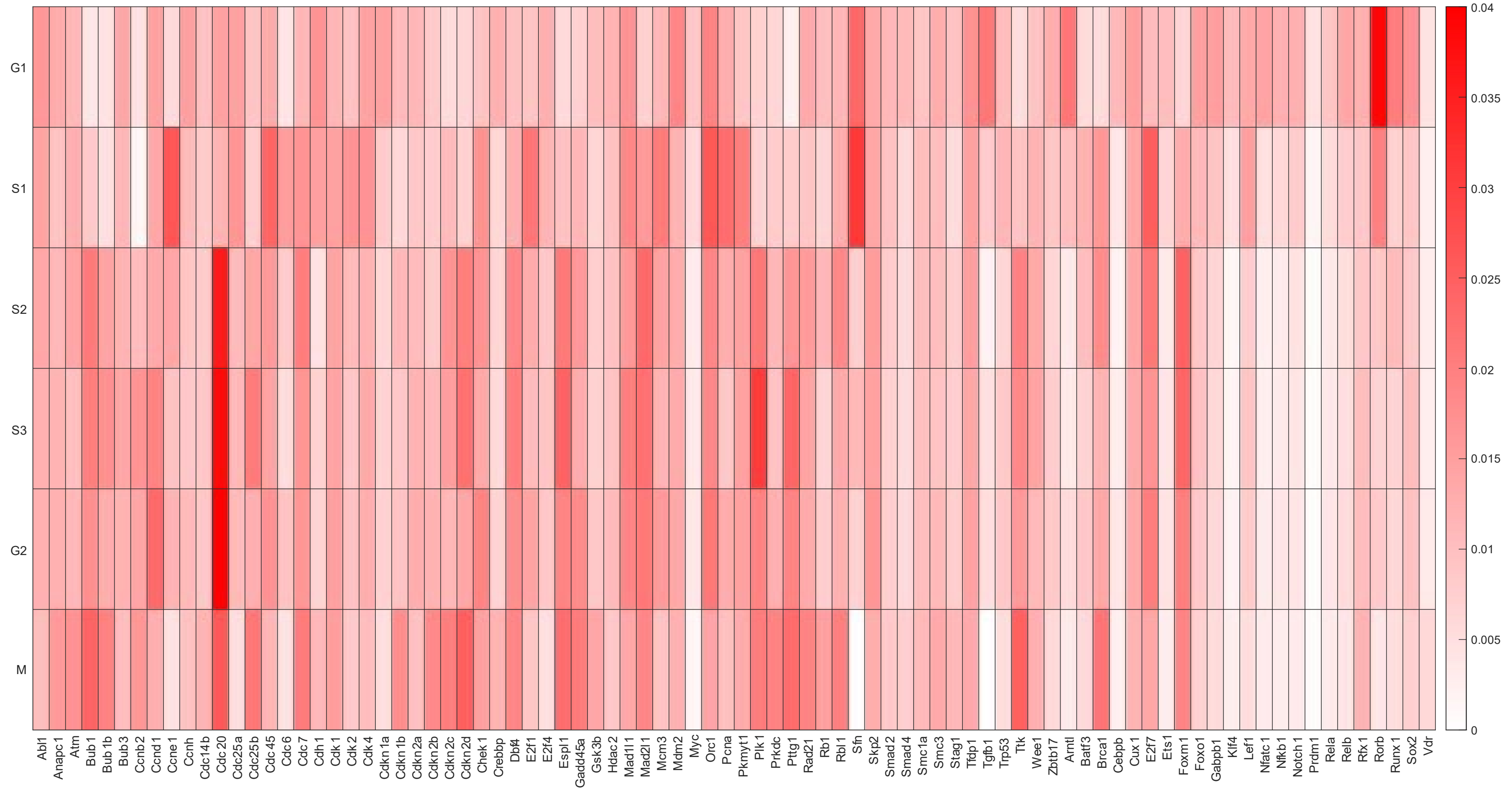

Figure S5: Stage-specific cybernetic weights.

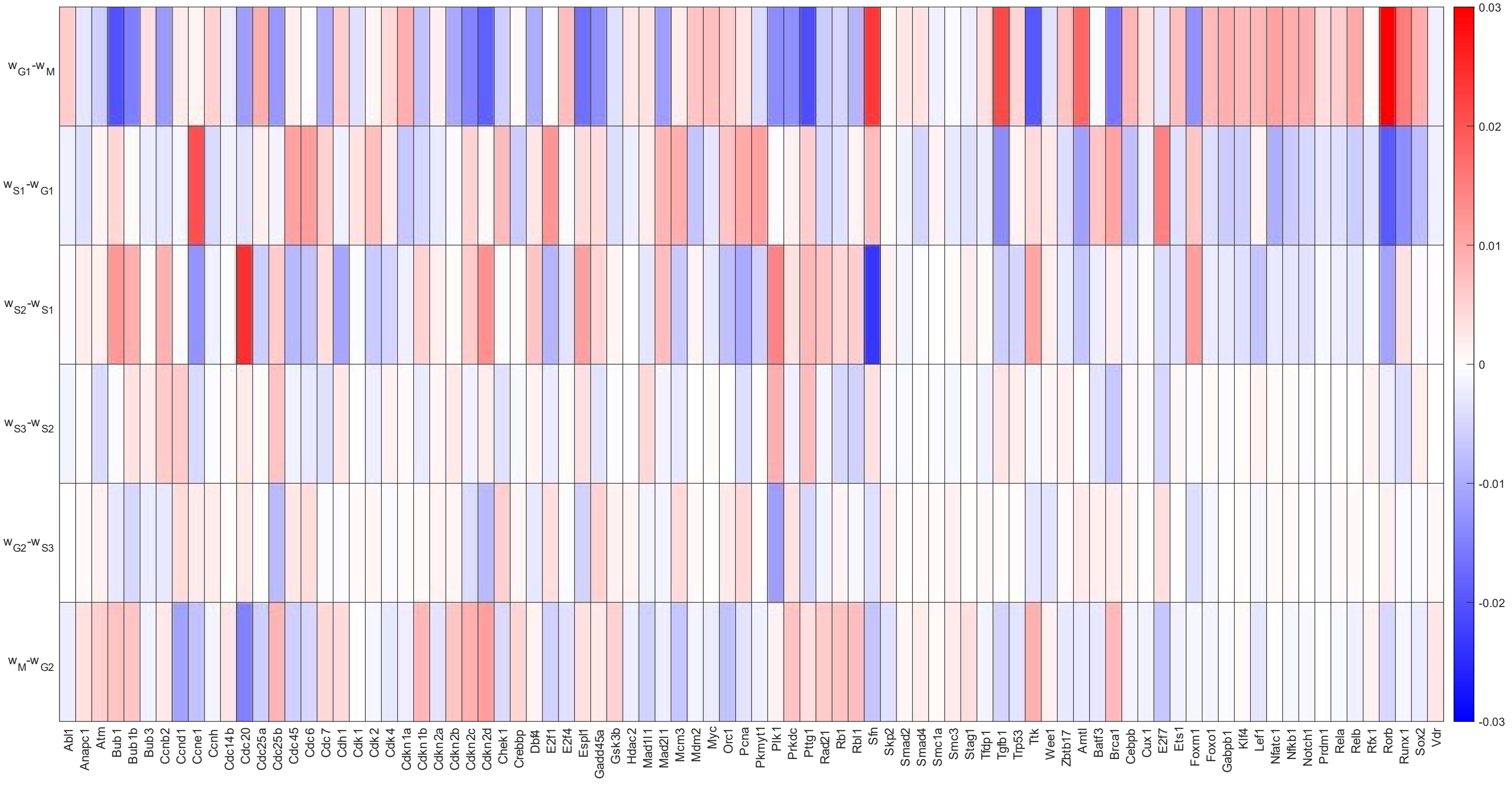

Figure S6: Cybernetic weight differences between adjacent stages.

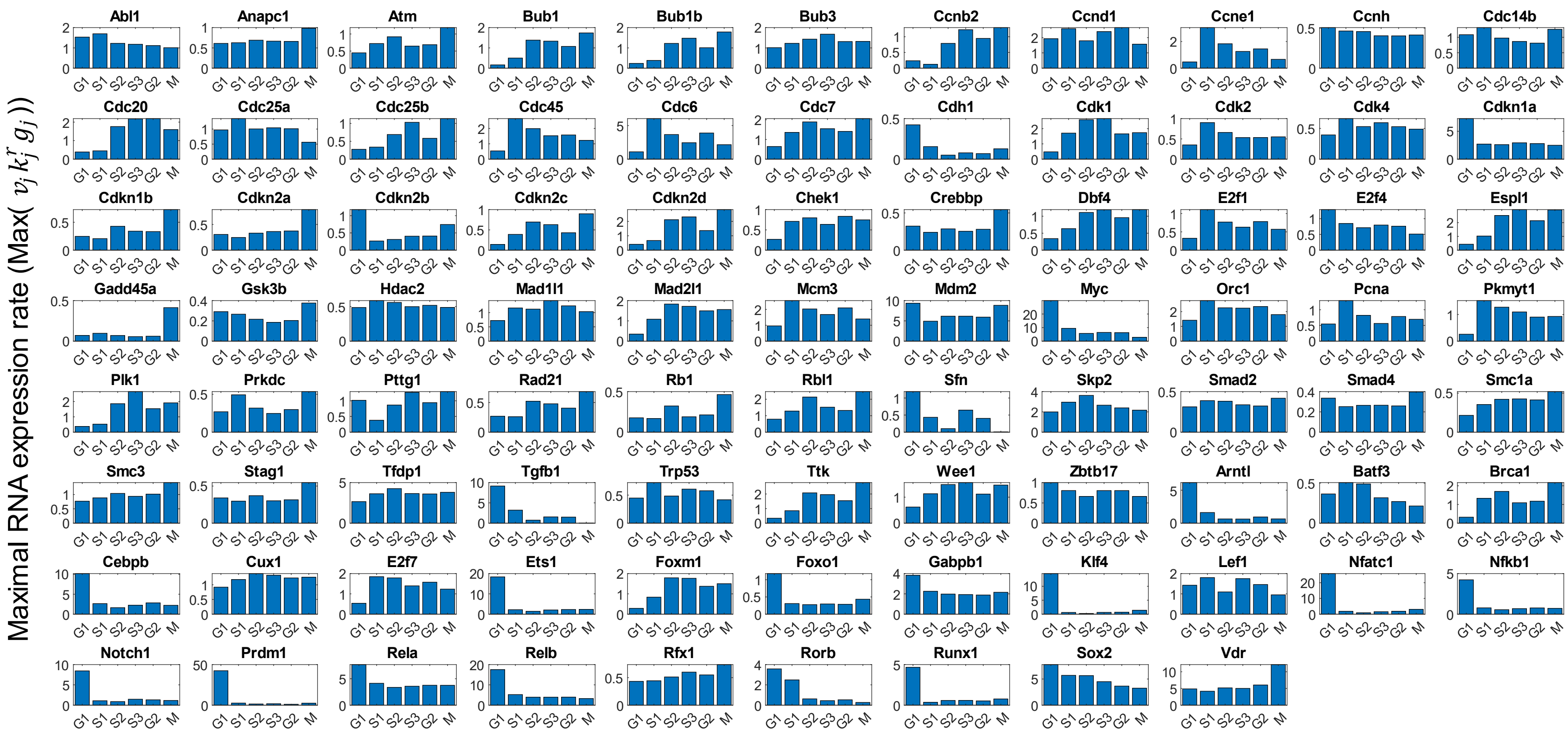

Figure S7: Stage-specific Maximal RNA expression rate (Max($v_j k_j^r g_j$))
